# Thyroid hormones increase stomach goblet cell numbers and mucin expression during indomethacin induced ulcer healing in Wistar rats

**DOI:** 10.1186/s13044-018-0050-0

**Published:** 2018-05-25

**Authors:** Jackline Namulema, Miriam Nansunga, Charles Drago Kato, Muhammudu Kalange, Samuel Babafemi Olaleye

**Affiliations:** 1 Department of Physiology, Faculty of Biomedical Sciences, Kampala International University, P.O BOX 71, Ishaka, Bushenyi Uganda; 2 Department of Immunology and Microbiology, Faculty of Biomedical Sciences, Kampala International University, P.O BOX 71, Ishaka, Bushenyi Uganda; 3 Laboratory for Gastrointestinal Secretion and Inflammation Research, Department of Physiology, College of Medicine, University of Ibadan, Ibadan, Nigeria; 4 Department of Physiology, Faculty of Biomedical Sciences, St Augustine International University, P.O BOX 88, Kampala, Uganda; 5 School of Biosecurity, Biotechnical & Laboratory Sciences, College of Veterinary Medicine, Animal Resources and Biosecurity, Makerere University, P.O Box 7062, Kampala, Uganda

**Keywords:** Thyroid hormones, Goblet cells, Mucins, Indomethacin, Ulcer healing

## Abstract

**Background:**

Gastric ulcers are mucosal discontinuities that may extend into the mucosa, submucosa or even deeper. They result from an imbalance between mucosal aggressors and protective mechanisms that include the mucus bicarbonate layer. Thyroid hormones have been shown to accelerate gastric ulcer healing in part by increasing the adherent mucus levels. However, the effects of thyroid hormones on goblet cell numbers and expression of neutral and acidic mucins during ulcer healing have not been investigated.

**Methods:**

Thirty six adult male Wistar rats were randomly divided into six groups each with six animals. Group 1 (normal control) and group 2 (negative control) were given normal saline for eight weeks. Groups 3 and 4 were given 100 μg/kg per day per os of thyroxine so as to induce hyperthyroidism. Groups 5 and 6 received 0.01% (*w*/*v*) Propylthiouracil (PTU) for 8 weeks so as to induce hypothyroidism. After thyroid hormonal levels were confirmed using radioimmunoassay and immunoradiometric assays, ulcer induction was done using 40 mg/kg intragastric single dose of Indomethacin in groups 2, 3 and 5. Stomachs were extracted after day 3 and 7 of ulcer induction for histological examination. Histochemistry was carried out using Periodic Acid Shiff and Alcian Blue. The number of acidic and neutral goblet cells were determined by counting numbers per field. Mucin expression (%) was determined using Quick Photo Industrial software version 3.1.

**Results:**

The numbers of neutral goblet cells (cells/field) increased significantly (*P* < 0.05) in the ulcer+thyroxine (14.67 ± 0.33), thyroxine (17.04 ± 1.71) and ulcer+PTU (12.89 ± 1.06) groups compared to the normal control (10.78 ± 1.07) at day 3. For the acidic goblet cells, differences between treatment groups were more pronounced at day 7 between the ulcer+thyroxine (22.56 ± 1.26) and thyroxine (22.89 ± 0.80). We further showed that percentage expression of both neutral and acidic mucins was significantly higher in the ulcer+thyroxine (9.23 ± 0.17 and 6.57 ± 0.35 respectively) and thyroxine groups (9.66 ± 0.21 and 6.33 ± 0.38 respectively) as compared to the normal control group (4.08 ± 0.20 and 4.38 ± 0.11 respectively) at day 3 after ulcer induction.

**Conclusion:**

This study confirms the role played by thyroid hormones in healing of indomethacin induced gastric ulcers. The study further demonstrates increased numbers of both neutral and acidic goblet cells and the increase in expression of both neutral and acidic mucins during healing of indomethacin induced ulcers.

**Electronic supplementary material:**

The online version of this article (10.1186/s13044-018-0050-0) contains supplementary material, which is available to authorized users.

## Background

Stomach or peptic ulcers are the most common gastrointestinal tract ailments that affect half of the world’s population [1]. The highest burden of this disease both in morbidity and mortality occurs in developing countries [2]. In Uganda, the rate of death from these ulcers is 2.81 per 100,000 individuals [3]. An ulcer of the gastrointestinal tract is defined as a discontinuity in the muscularis mucosae into the submucosa or deeper [4]. Along the length of the gastrointestinal tract, ulcers commonly occur in the stomach and the duodenum [5]. They are due to an imbalance between the protective and aggressive mechanisms including pepsin, *Helicobacter pylori* infection and gastric acid [6].

Normally, the epithelial cells of the stomach are protected from various aggressive factors by a mucus-bicarbonate-phospholipid barrier which conserves the continuity of the surface epithelium [7, 8] thus preventing the ulcers from occurring. The adherent mucus layer, a very important component of the barrier is secreted by surface epithelial cells (goblet cells) [9–11]. The mucous covers the stomach epithelial cells and serves as the first line of defense against mucosal aggressors [10, 12].The mucus is formed by gel-forming mucin glycoproteins (MUCs) and water [13]. In order for the levels of mucus to increase after any stress, the cell surface mucins first detect the changes in the external environment and signal these changes to the goblet cell [14]. This is followed by increase in the adherent mucus levels that promote ulcer healing.

Four mucins are found in the stomach MUC1, MUC4, MUC5AC and MUC6 [15]. These mucins are histochemically classified into acidic and neutral mucins [16, 17]. The expression of the different mucin types depends on the type of mucosal aggressor [13, 18].

Ulcer healing depends on the removal of the aggressive mechanisms and increase in the protective mechanisms [19, 20]. Since the adherent mucus layer is the first line of defenses against ulcers [10], it follows that mucus secretion increases as ulcer healing progresses. In secretory organs and tissues, thyroid hormones have been associated with increased body fluid secretion [21]. In the stomach, these hormones have been shown to accelerate ulcer healing when given before or at the beginning of the stress [21]. Ulcer healing has been associated with an increase in adherent mucus content [22], accelerated reepithelization [23] and angiogenesis [24]. The increase in the mucus levels could be attributed to the increase in the number of goblet cells or in their individual secretion rate. Therefore, the present study aimed at determining the effect of thyroid hormones on goblet cell numbers and expression of both neutral and acidic mucins in the stomach of wistar rats during healing of indomethacin induced ulcers.

## Methods

### Experimental design

Thirty six adult male Wistar albino rats with an average weight 180 ± 21.4 g were procured from the Department of Pharmacology, Kampala International University-Western Campus. The rats were housed in standard rat cages in the animal house at the Department of Pharmacology with access to food and water for two weeks before the beginning of the experiment. Animals were grouped into six groups each having six rats that were careful matched for weight (Additional file 1: Table S1). Group one (normal control) and group two (negative control) were given normal saline for eight weeks. Hyperthyroidism was induced in group three and group four by administering thyroxine (Leehpl ventures, India) at a dose of 100 μg/kg per day per os for eight weeks [25]. Hypothyroidism was induced in group five and group six through the administration of Propylthiouracil, PTU (Macleod, India) as described previously [26]. Thyroid hormone levels were confirmed using radioimmunoassay for thyroxine and triiodothyronine and immunoradiometric assay for thyroid stimulating hormone as described previously [23] (results shown in Additional file 2: Table S2). After an overnight fast but with access to water, ulcers were induced in groups 2, 3 and 5 using 40 mg/kg intragastric single dose of Indomethacin (Sun Pharma, India) as previously described [27]. After ulcer induction, treatment with respective drugs were stopped.

### Histochemistry studies

From each group, three rats were sacrificed on day three and the remaining three rats sacrificed at day 7 after ulcer induction under diethyl ether anaesthesia. The abdomens were opened along the greater curvature and the stomach excised. The stomachs were rinsed with saline water and later fixed in 10% formalin. Prior to any staining procedures, sections were cut at 5 μm using a microtome and placed on glass slides. The sections were deparaffinised in xylene and rehydrated in a graded alcohol series. Six slides were prepared for each rat; three for acidic goblet cells and expression of acidic mucins and the other three for neutral goblet cells and expression of neutral mucins.

Determination of neutral goblet cells and mucins was by a method described by Forder et al. [28]. Briefly, following the deparaffinization and rehydration, the sections were subjected to mild acid hydrolysis to eliminate the contribution of acid residues. They were incubated in 0.5% Periodic Acid (PA) for 20 min and washed and incubated with Schiff’s reagent for 20 min. After rinsed in tap water for 10 min, were dehydrated and then mounted on a light microscope.

Determination of acidic goblet cells and mucins was by a method described by Uni et al. [29]. Briefly, following the deparaffinization and rehydration, sections were incubated in 3% acetic acid for 3 min and then Alcian Blue solution (1% in 3% acetic acid, pH 2.5). Slides were rinsed in water, dehydrated and mounted on a light microscope.

### Image analysis

After examination, selected slides were photographed using a digital camera (Canon, China) mounted on a light microscope (Carl Zeiss, Germany). Images were acquired from the slides using Zoom browser Ex, version 2 Imaging software. Goblet cells were counted according to the method described by [30] with slight modification. Briefly, goblet cells that stained with Periodic Acid Schiff (PAS) and Alcian Blue (AB) were counted using a calibrated microscope in five randomly selected areas of the gastric mucosal tissue and presented as numbers per field. Counts were done in triplicate for each slide. The mean value was calculated for each group and represented as Mean ± SEM.

Determination of the levels of mucin expression was carried out using a method described by Nonose et al. [31]. The expression of neutral and acid mucins was quantified by means of computer-assisted image processing. The images selected were captured on a video camera that was coupled to an optical microscope. These images were processed and analyzed using the Quick PHOTO INDUSTRIAL software version 3.1. The software determined the color intensity in number of pixels in each field selected, and transformed the final data into percentage expressions by analyzed fields. The final value taken for each field measured in the segments was the mean of the values found from evaluating three different fields. Data was presented as Mean ± SEM in percentage.

### Statistical analysis

Statistical analysis was done using Graph pad Prisms Version 6. Mean values for the number of the goblet cells and mucin intensity (%) were obtained for each group of rats. Data was presented as Mean ± SEM (Standard Error of Mean). Within treatment groups comparisons were made for each group of rats at day 3 and 7 after ulcer induction using a Student’s t-test. Comparisons of the means across treatment groups were done using one way ANOVA followed by Tukey’s multiple comparison tests. Significant differences in the means for both statistical tests were considered at *P* < 0.05 level.

## Results

### Effect of thyroid hormones on ulcer healing

**Fig. 1:**
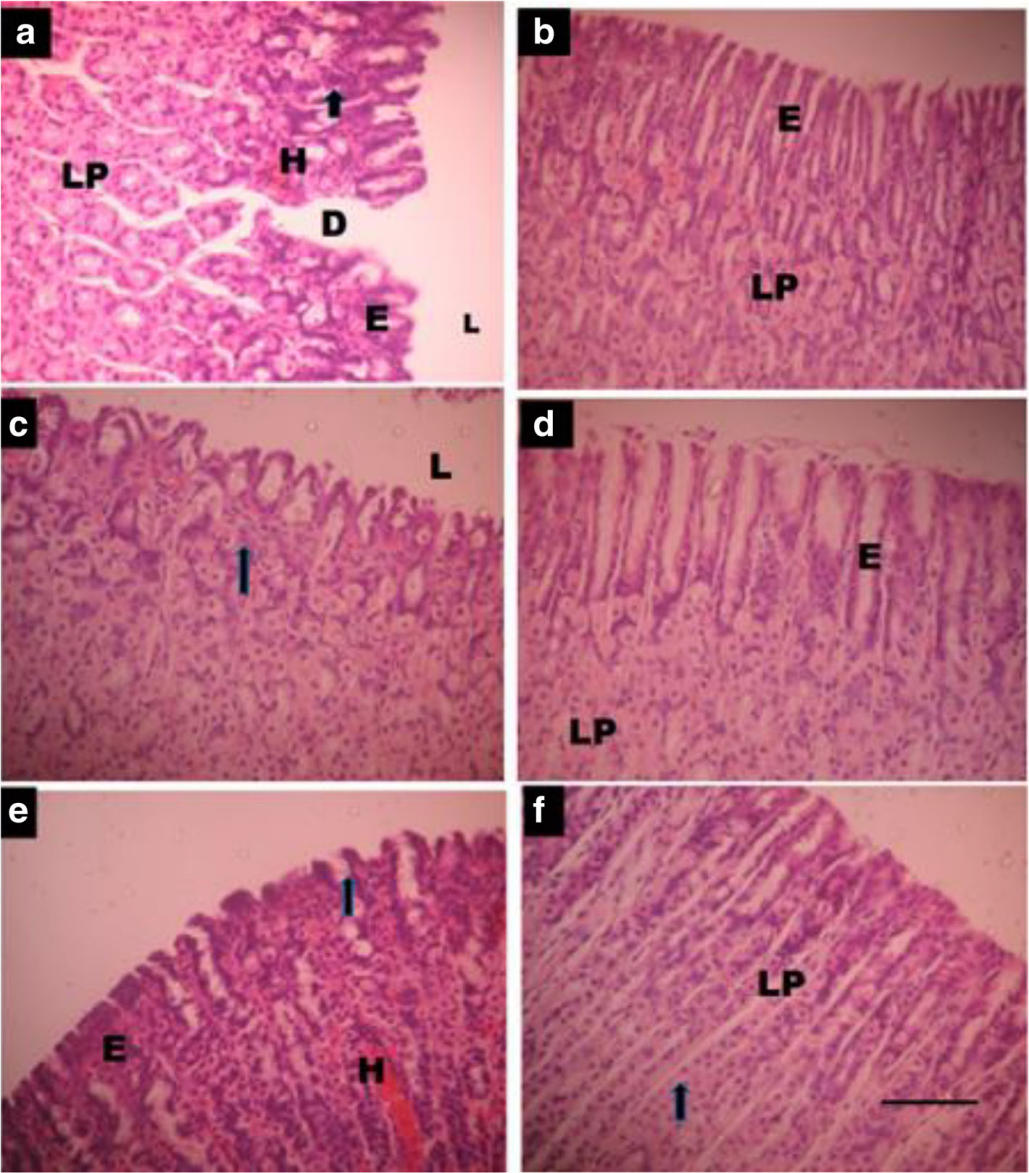
Effect of thyroid hormones on the healing of indomethacin induced gastric ulcers at days 3 (**a**, **c** and **e**) and 7 (**b**, **d** and **f**) after ulcer induction. Mucosal injury characterized by, discontinuity in the mucosa (**d**), lymphocyte infiltration (arrows) and erosions (R) in the epithelium (**e**) and lamina propria (LP), hemorrhage (H) can be seen in Plate **a** and **e**. **a** and **b**, ulcer+normal saline, **c** and **d**, ulcer+thyroxine and **e** and **f**, ulcer+PTU. L-lumen

In order to demonstrate ulcer induction and the effect of thyroid hormones on ulcer healing, the histopathology of the stomach was analyzed among experimental animals. At day 3 after ulcer induction, the results showed that the ulcer+normal saline and ulcer+PTU (hypothyroid) groups had regions of mucosal injury characterized by discontinuity of the mucosal epithelium, lymphocyte infiltration, erosions in the lamina propria, tissue hemorrhages and necrosis (Fig. 1). By day 7, there was marked improvement in the ulcer+normal saline group to levels comparable to the normal control. The ulcer+PTU group still had lymphocyte infiltration and erosions by day 7. In the ulcer+thyroxine group, there was minimal mucosa injury by day 3 and by day 7, the mucosal integrity was restored and comparable to that of the normal control group (Fig. 1).

### Effect of thyroid hormones on the number of goblet cells

In order to measure the effect of thyroid hormones on goblet cell numbers during ulcer healing, we enumerated the number of acidic and neutral goblet cells. The results showed that both neutral and acidic goblet cell numbers significantly differed across treatment groups at both day 3 (F _[5, 12]_ = 4.09, *P* = 0.02 and F _[5, 12]_ = 16.74 *P* < 0.0001 respectively) and day 7 (F _[5, 12]_ = 24.63, *P* < 0.0001 and F _[5, 12]_ = 20.19, *P* < 0.0001 respectively).

**Fig. 2:**
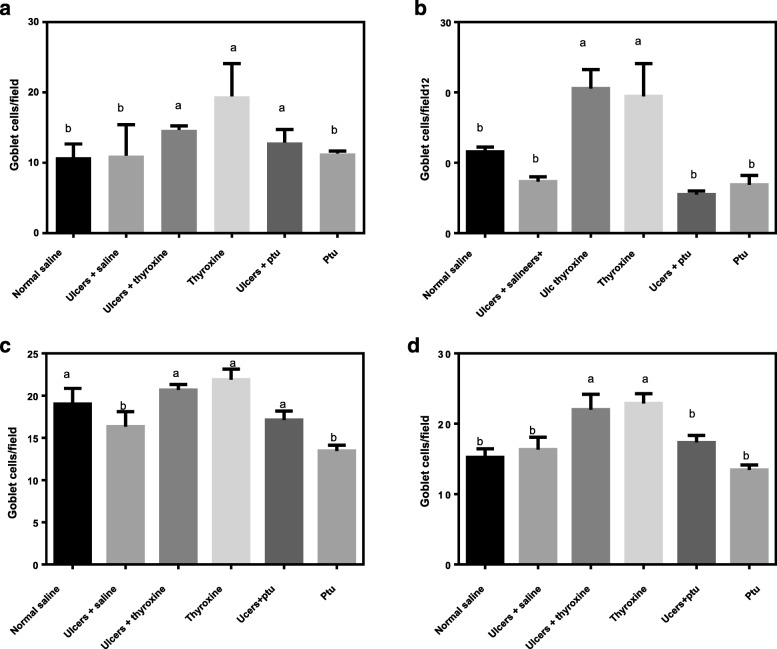
The effect of Thyroid hormones on the numbers of neutral (**a** and **b**) and acidic (**c** and **d**) goblet cells during healing of indomethacin induced gastric ulcers at day 3 (**a** and **c**) and 7 (**b** and **d**) after ulcer induction respectively. PTU-Propylthiouracil. Different superscripts indicate significant differences

The numbers of neutral goblet cells (cells/field) increased significantly (*P* < 0.05) in the ulcer+thyroxine (14.67 ± 0.33), thyroxine (17.04 ± 1.71) and ulcer+PTU (12.89 ± 1.06) groups compared to the normal control (10.78 ± 1.07) at day 3 (Fig. 2a). However, by day 7, only the ulcer+thyroxine (20.07 ± 1.56) and thyroxine (19.44 ± 2.70) groups showed significant differences (*P* < 0.05) compared to the normal control (11.56 ± 0.40) (Fig. 2b). For the acidic goblet cells, differences between treatment groups were more pronounced at day 7 (Fig. 2d) between the ulcer+thyroxine (22.56 ± 1.26) and thyroxine (22.89 ± 0.80) as compared to the normal control (15.23 ± 0.69). On comparison of goblet cells at day 3 and 7 within the treatment groups, the number of neutral goblet cells was significantly increased in the ulcer+thyroxine (*P* < 0.02) group with no significant differences in the other groups. There were no significant differences in numbers of acidic goblet cells within treatment groups at both days (Table 1).

**Table 1:** Comparison of goblet numbers at day 3 and 7 during ulcer healing

Treatment	*N*	Neutral			Acidic		
		Day		*P*-value	Day		*P*-value
		3	7		3	7	
Normal control	6	10.78 ± 1.09	10.62 ± 0.9	0.32	19 1.07	15.23 0.69	0.41
Ulcer + Normal saline	6	11.00 ± 2.55	7.33 ± 0.38	0.23	16.33 1.02	17.33 0.39	0.37
Ulcer + Thyroxine	6	14.67 ± 0.33	20.56 ± 1.56	0.02*	20.67 0.38	22.56 1.26	0.07
Ulcer + PTU	6	8.00 ± 1.06	11.78 ± 0.40	0.39	17.11 0.62	17.33 0.58	0.16

Asterisks indicate significant differences at *P*<0.05

### Effect of thyroid hormones on the expression of neutral and acidic mucins during ulcer healing

**Fig. 3:**
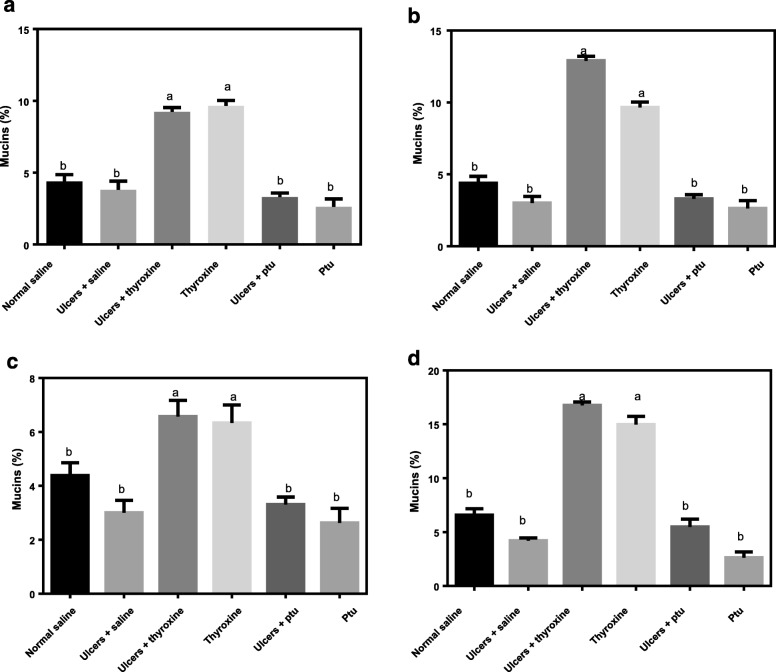
Effect of Thyroid Hormones on the expression of neutral and acidic mucins during ulcer healing. **a** and **b** show expression of neutral mucins at day 3 and 7 after ulcer induction respectively. **c** and **d** show expression of acidic mucins at day 3 and 7 after ulcer induction respectively. Different superscripts indicate significant differences between the treatment groups

In order to determine the effect of thyroid hormones on the expression of neutral and acidic mucins during ulcer healing, the intensity of the stains as an indicator of mucin intensity was measured. The results showed that thyroid hormones significantly increased the expression of both neutral and acidic mucins at both day 3 (F _(5, 12)_ = 308, *P* < 0.0001 and F _(5, 12)_ = 32, *P* value < 0.0001 respectively) and day 7 (F _(5, 12)_ = 308.5, *P* < 0.0001 and F _(5, 12)_ = 32.65, *P* < 0.0001 respectively) after ulcer induction with indomethacin. Comparisons between the different groups showed that the percentage expression of both neutral (Fig. 3a) and acidic (Fig. 3c) mucins was significantly higher in the ulcer+thyroxine (9.23 ± 0.17 and 6.57 ± 0.35 respectively) and thyroxine groups (9.66 ± 0.21 and 6.33 ± 0.38 respectively) as compared to the normal control group (4.08 ± 0.20 and 4.38 ± 0.11 respectively) at day 3 after ulcer induction. By day 7 after ulcer induction, the expression of both neutral (Fig. 3b) and acidic (Fig. 3d) mucins in the ulcer+thyroxine (12.9 ± 0.18 and 16 ± 1.26 respectively) and thyroxine (9.73 ± 0.21 and 14.89 ± 0.80 respectively) treated groups remained significantly elevated as compared to the normal control (4.58 ± 0.28 and 7.23 ± 0.69 respectively). Comparison of the number of goblet cells within treatment groups at days 3 and 7 after ulcer induction showed a significant increase in the expression of neutral goblet cells in the ulcer+saline (3.00 ± 0.35 at day 3 to 3.80 ± 0.27 at day 7, *P* < 0.0004). In addition, to the ulcer+thyroxine group (9.23 ± 0.21 at day 3 to 9.73 ± 0.21 at day 7, *P* < 0.00001). Only the ulcer+thyroxine (4.70 ± 0.28 at day 3 to 16.97 ± 0.18 at day 7, *P* < 0.00001) group showed a significant increase in the expression of acidic mucins (Table 2).

**Table 2:** Comparison of the expression of neutral and acidic mucins at day 3 and 7

Treatment	N	Neutral mucins			Acidic mucins		
		Day		*P*-value	Day		*P*-value
		3	7		3	7	
Normal control	6	4.08 ± 0.20	4.58 ± 0.28	0.04	12.81 ± 0.19	7.23 ± 0.69	0.07
Ulcer + Normal saline	6	3.00 ± 0.35	3.80 ± 0.27	0.0004*	9.66 ± 0.22	4.73 ± 0.43	0.08
Ulcer + Thyroxine	6	9.23 ± 0.21	9.73 ± 0.21	0.00001*	4.70 ± 0.28	16.97 ± 0.18	0.01*
Ulcer + PTU	6	3.31 ± 0.16	2.31 ± 0.11	0.06	3.26 ± 0.16	5.87 ± 0.42	0.12

Asterisks indicate significant differences at *P* < 0.05

## Discussion

The results of the study showed that increased thyroid hormone levels accelerated healing of Indomethacin induced ulcers by day 7 after ulcer induction. This gastroprotective effect was indicated by the clearance of inflammatory cells and restoration of mucosal integrity by day 7 after ulcer induction in the ulcer+thyroxine (hyperthyroid) group yet these persisted in the ulcer +PTU (hypothyroid) group compared to the normal (euthyroid) control. This is in agreement with other studies that showed that high levels of thyroid hormones accelerated the healing of acetic acid [23, 25] and indomethacin induced ulcers when given before or after induction of the stress [21]. The faster clearance of inflammatory cells by thyroid hormones indicates that thyroid hormones speed up the inflammatory phase of gastric ulcer healing. The early clearance of these cells by thyroid hormones provides room for their replacement by fibroblasts and angiogenesis, processes that are also promoted by thyroid hormones [32]. Thyroid hormones also increase blood flow [33] to supply essential nutrients that promote ulcer healing. The ulcer+thyroxine and thyroxine groups showed significant increase in the number of goblet cells compared to the normal control by both days after ulcer induction. This is in agreement with other studies that have showed thyroid hormones to increase the rate of differentiation [34] and mitotic activity in different parts of the body [35] and that the hormones promote re-epithelization of the gastric mucosa during ulcer healing [25]. Since goblets cells are part of the cells that make up the gastric mucosa, it follows that thyroid hormones also increase their numbers.

The results of the study also showed that there was an increase in the expression of neutral mucins in the ulcer+thyroxine and thyroxine groups as compared to the normal control group at day 3 after ulcer induction. However, the increase in neutral mucins was more pronounced. This study confirms the findings that thyroid hormones increase the rate of activity of almost all cells [36], including the goblet cells. This is also in line with other studies that have showed that thyroid hormones increase the levels of adherent mucus during ulcer healing [23]. Thyroid hormones have been associated with a decrease in the pH of luminal contents that prevents exacerbation of the ulcers [22]. This could be attributed to their ability to increase the secretion of neutral mucins since they neutralize the acid thus reducing the pH [10]. On the other hand, the increase in the secretion of acidic mucins is important for the healing of the ulcers to progress since they contain chelating groups [29] and therefore act as antibacterial and antiviral agents [28]. Acidic mucins are also thick and viscous and thus form the major part of the protective mucus layer for both protection and lubrication [37], factors that are associated with healing of gastric ulcers. Studies have shown the stomach contains only two secreted mucins; MUC5AC (acidic) and MUC6 (neutral) [38]. It follows that thyroid hormones probably increase the expression of both gastric secreted mucins during ulcer healing.

## Conclusion

We show in here that thyroid hormones accelerate healing of Indomethacin induced gastric ulcers by increasing the numbers of both neutral and acidic goblets cells and the expression of both neutral and acidic mucins during healing of indomethacin induced ulcers in wistar rats. Therefore, in order to accelerate healing of indomethacin induced gastric ulcers, thyroid hormones increase the number of neutral and acidic goblet cells which is followed by an increase in the expression of both neutral and acidic mucins. Since in study histochemistry was used to show the expression of the mucins, other studies should be done using advanced molecular biology techniques to ascertain the effect of thyroid hormones on the exact mucin levels of the different types of stomach mucins during healing of indomethacin induced ulcers.

## Additional files

Additional file 1: **Table S1.** Treatments administered to the different groups. (DOCX 12 kb)

Additional file 2: **Table S2.** Thyroid hormone levels after 8 weeks of treatment. (DOCX 12 kb)

## Abbreviations

IRMA: Immunoradiometric assay
LMICs: Low and Middle Income countries
MUC: Mucin
NSAIDs: Non-steroid anti-inflammatory drugs
PTU: Propylthiouracil
PUD: Peptic Ulcer Disease
RIA: Radioimmunoassay
SEM: Standard Error of Mean
T3: Triiodothyronine
T4: Thyroxine
THs: Thyroid Hormones
TSH: Thyroid Stimulating Hormone
