# Supplementary material for: Thyroid hormones increase stomach goblet cell numbers and mucin expression during indomethacin induced ulcer healing in Wistar rats
Source: Thyroid Res. 2018 May 25;11:6. doi: 10.1186/s13044-018-0050-0 (PMC5970504; doi:10.1186/s13044-018-0050-0)
Supplement: Supplementary file 1 — Table S1. Treatments administered to the different groups. (DOCX 12 kb) [file 13044_2018_50_MOESM1_ESM.docx]

**S1 Table:** Treatments administered to the different groups

| **Groups** | **N** | **Treatment** |
| --- | --- | --- |
| Group 1 (Normal control) | 6 | 2 ml normal saline |
| Group 2 (Negative control) | 6 | Ulcer induction + 2 ml of normal saline |
| Group 3 | 6 | Ulcer induction + 100µg/kg thyroxine |
| Group 4 | 6 | 100µg/kg thyroxine |
| Group 5 | 6 | Ulcer induction + 0.01% (w/v) Propylthiouracil |
| Group 6 | 6 | 0.01% (w/v) Propylthiouracil |
