# Supplementary material for: Thyroid hormones increase stomach goblet cell numbers and mucin expression during indomethacin induced ulcer healing in Wistar rats
Source: Thyroid Res. 2018 May 25;11:6. doi: 10.1186/s13044-018-0050-0 (PMC5970504; doi:10.1186/s13044-018-0050-0)
Supplement: Supplementary file 2 — Table S2. Thyroid hormone levels after 8 weeks of treatment. (DOCX 12 kb) [file 13044_2018_50_MOESM2_ESM.docx]

**S2 Table:** Thyroid hormone levels after 8 weeks of treatment

| **Hormones** | **Thyroxine treated** | **Propylthiouracil treated** | **Normal control** | **pvalue** |
| --- | --- | --- | --- | --- |
| Triiodothyronine (T3) | 13.01±0.31^a^ | 1.05±.05^c^ | 3.85±0.12^b^ | P< 0.0001 |
| Thyroxine (T4) | 11.87±0.37^a^ | 1.12±0.08^c^ | 3.8±0.16^b^ | P< 0.0001 |
| Thyroid stimulating hormone (TSH) | 0.69±0.06^b^ | 2.18±0.07^a^ | 1.00±0.04^b^ | P< 0.0001 |
| Superscript letters ^a, b, c^ indicate significant differences across treatments | | | |  |
